# Dietary fat-associated osteoarthritic chondrocytes gain resistance to lipotoxicity through PKCK2/STAMP2/FSP27

**DOI:** 10.1038/s41413-018-0020-0

**Published:** 2018-07-06

**Authors:** Sung Won Lee, Jee Hyun Rho, Sang Yeob Lee, Won Tae Chung, Yoo Jin Oh, Jung Ha Kim, Seung Hee Yoo, Woo Young Kwon, Ju Yong Bae, Su Young Seo, Hokeun Sun, Hye Young Kim, Young Hyun Yoo

**Affiliations:** 1 Department of Rheumatology, Dong-A University College of Medicine, Busan, Republic of Korea; 2 Department of Anatomy and Cell Biology, Dong-A University College of Medicine and Mitochondria Hub Regulation Center, Busan, Republic of Korea; 3 Department of Microbiology, Dong-A University College of Medicine, Busan, Republic of Korea; 4 Department of Statistics, Pusan National University, Busan, Republic of Korea

## Abstract

Free fatty acids (FFAs), which are elevated with metabolic syndrome, are considered the principal offender exerting lipotoxicity. Few previous studies have reported a causal relationship between FFAs and osteoarthritis pathogenesis. However, the molecular mechanism by which FFAs exert lipotoxicity and induce osteoarthritis remains largely unknown. We here observed that oleate at the usual clinical range does not exert lipotoxicity while oleate at high pathological ranges exerted lipotoxicity through apoptosis in articular chondrocytes. By investigating the differential effect of oleate at toxic and nontoxic concentrations, we revealed that lipid droplet (LD) accumulation confers articular chondrocytes, the resistance to lipotoxicity. Using high fat diet-induced osteoarthritis models and articular chondrocytes treated with oleate alone or oleate plus palmitate, we demonstrated that articular chondrocytes gain resistance to lipotoxicity through protein kinase casein kinase 2 (PKCK2)—six-transmembrane protein of prostate 2 (STAMP2)—and fat-specific protein 27 (FSP27)-mediated LD accumulation. We further observed that the exertion of FFAs-induced lipotoxicity was correlated with the increased concentration of cellular FFAs freed from LDs, whether FFAs are saturated or not. In conclusion, PKCK2/STAMP2/FSP27-mediated sequestration of FFAs in LD rescues osteoarthritic chondrocytes. PKCK2/STAMP2/FSP27 should be considered for interventions against metabolic OA.

## Oil droplets protect cartilage from toxic fatty acids

Cartilage tissue deals with the stress of exposure to free fatty acids by sequestering the toxic molecules into sub-cellular oil droplets. Young Hyun Yoo from Dong-A University College of Medicine in Busan, South Korea, and coworkers exposed rat cartilage cells to increasing levels of a fatty acid called oleate, a by-product of fat metabolism, and observed that the accumulation of oil droplets conferred resistance to oleate-induced toxicity. In these rat cells and in experiments involving mouse models of osteoarthritis fed a high-fat diet, the researchers then identified three of the protective proteins needed for cartilage tissue to properly quarantine fatty acids into oil droplets. Those proteins — and their connected regulatory networks — could now serve as drug targets for treating metabolic syndrome-associated osteoarthritis.

## Introduction

Osteoarthritis (OA) is a multifactorial disease characterised by degradation of the extracellular matrix and the destruction of articular cartilage. Because chondrocytes are the only resident cells in human articular cartilage and cell matrix turnover in cartilage is solely dependent on these cells, the death of articular chondrocytes is generally considered to play a central role in OA cartilage destruction. To date, stimuli involved in chondrocyte death and their signalling pathways have been highlighted as pathogenetic factors leading to joint cartilage degradation.^1–5^

OA is now considered a complex disease with different clinical subtypes. Among these subtypes, metabolic OA is distinguished from other subtypes by the presence of obesity or metabolic syndrome, low-grade systemic inflammation, earlier onset and a faster progression.^6^ However, the concept of the disease as a 'wear-and-tear disease', which is traditionally accepted for the pathophysiology of OA, does not seem to account for the cartilage destruction in metabolic OA. Moreover, joint overload is unable to explain strong epidemiological data, demonstrating the association between obesity and hand OA,^7^ although obese patients with metabolic syndrome have an increased risk of knee OA compared with that of obese patients without metabolic syndrome.^8^ Thus, systemic factors must be involved in the pathogenesis of OA. Recent studies led to the discovery of pro-inflammatory cytokines and adipokines produced by the adipose tissue as central contributors to metabolic OA of the hand and potentially other locations.^7^

Lipid imbalance is a key metabolic alteration associated with metabolic syndrome and obesity. In hyperlipidaemic states, lipids abnormally accumulate in non-adipose tissues. Articular chondrocytes, unlike most other cells, are characterised by their substantial stores of lipid deposits.^9–13^ A previous study demonstrated the existence of a marked and graded increase in the total fatty acids in articular cartilage from OA joints.^13^ In hyperlipidaemic states, the accumulation of excess lipids in non-adipose tissues exerts lipotoxicity, leading to cell dysfunction and/or cell death. Free fatty acids (FFAs), which are elevated with metabolic syndrome or obesity, are considered the principal offender exerting lipotoxicity and inducing apoptosis, insulin resistance and inflammation. Thus, it seems readily presumable that the accumulation of FFAs contributes to OA pathogenesis. However, the causal relationship between FFAs and OA pathogenesis has only recently been demonstrated. Few previous studies demonstrated that dietary fat induced osteoarthritis.^14,15^ In addition, a recent study demonstrated that palmitate, but not oleate, has a pro-apoptotic effect on interleukin 1 beta (IL-1-β)-stimulated articular chondrocytes.^16^ However, the molecular mechanism by which FFAs exert lipotoxicity remains largely unknown.

In this study, we investigated the molecular mechanism by which FFAs exert pro-apoptotic effects by focusing on the following urgent questions related to FFA accumulation-associated OA. First, what is the underlying mechanism by which FFA exerts lipotoxicity in articular chondrocytes? Second, what is the meaning of the differential effect of saturated FFA and unsaturated FFA on chondrocyte death in terms of OA pathophysiology? Third, with the assumption that articular chondrocytes struggle to survive against FFA-induced lipotoxicity, what is the mechanism by which articular chondrocytes survive under the effects of FFA accumulation?

Our results demonstrated that protein kinase casein kinase 2 (PKCK2)‑, six-transmembrane protein of prostate 2 (STAMP2)‑ and fat-specific protein 27 (FSP27)-mediated sequestration of FFAs in lipid droplets (LDs) confers articular chondrocytes the ability to resist lipotoxicity.

## Results

### High-fed diet (HFD) accelerates the onset of OA

**Fig. 1:**
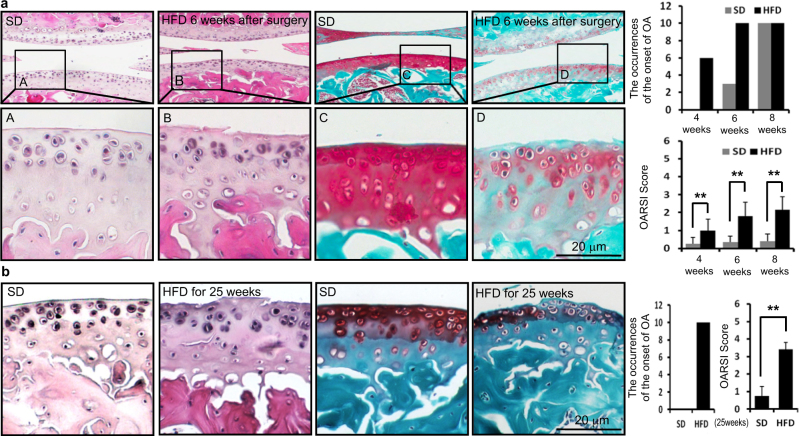
HFD accelerates the onset of OA. The onset of OA was determined by the irregular surface and the disappearance of surface layer cells from tissues stained with H&E and reduced Safranin O. Scale bars, 20 μm. **a** Mice fed an SD or an HFD for 12 weeks were subjected to surgery for experimental OA, and after 4 weeks (*n* = 10 for each diet), 6 weeks (*n* = 10 for each diet) and 8 weeks (*n* = 10 for each diet), the cartilage was observed. Representative histologic findings show that characteristic OA findings were observed 6 weeks after surgery in mice fed an HFD but not in mice fed an SD. The graph shows the occurrence of the onset of OA among mice fed an SD or an HFD during three different weeks. The dietary variable showed a significant difference between SD and HFD groups based on the likelihood ratio test (*P* < 0.01) from Poisson regression analysis. OA was more severe in HFD-fed mice compared to SD-fed mice as determined by OARSI scoring. **P < 0.01 according to Scheffe’s test. **b** Mice were fed an SD or an HFD for 25 weeks, and the cartilage was observed. Characteristic OA findings were observed in all mice fed an HFD (*n* = 10) but not in mice fed an SD (*n* = 10). The graph shows the occurrences of the onset of OA among 10 mice for the SD and HFD groups. There was a significant difference between SD and HFD feeding (*P* < 0.01) according to the chi-squared test. OA was more severe in HFD-fed mice compared to SD-fed mice as determined by OARSI scoring. **P < 0.01 according to Scheffe’s test

Using two experimental mouse OA models, we examined whether an HFD accelerated the onset of OA. The onset of OA was determined by histological findings characteristic of OA, such as an irregular surface, the disappearance of surface layer cells and reduced Safranin O staining. First, mice fed a standard diet (SD) or an HFD for 12 weeks were subjected to surgery for experimental OA, and after the indicated time, cartilages were histologically observed. Among various surgical OA models, we used the anterior cruciate ligament transection (ACLT) model, which is the most commonly used surgical model in OA research today. We could observe the onset of OA at 4 weeks (6/10) or at least 6 weeks (10/10) after surgery in mice fed an HFD. In contrast, these findings were not observed in any mouse fed an SD 4 weeks after surgery, while these findings were observed at 6 weeks (3/10) and 8 weeks (10/10) after surgery in mice fed an SD. There was a statistically significant difference between the SD and HFD based on the likelihood ratio test of the Poisson regression model (*P* = 0.000 000 000 030 05). OA was also more severe in HFD-fed mice compared to SD-fed mice as determined by Osteoarthritis Research Society International (OARSI) scoring (Fig. 1a). Furthermore, we examined the onset of OA findings in mice fed an SD or an HFD for 25 weeks without surgery for experimental OA. We observed characteristic OA findings in all mice fed an HFD (10/10) but not in any mouse fed an SD (0/10). Indeed, there was statistical significance for the probability of OA findings between mice fed an SD or an HFD based on the *χ*^2^ test (*P* = 0.019 92). OA was also more severe in HFD-fed mice compared to SD-fed mice as determined by OARSI scoring (Fig. 1b). These findings indicate that long time HFD feeding itself induces the onset of OA.

**Fig. 2:**
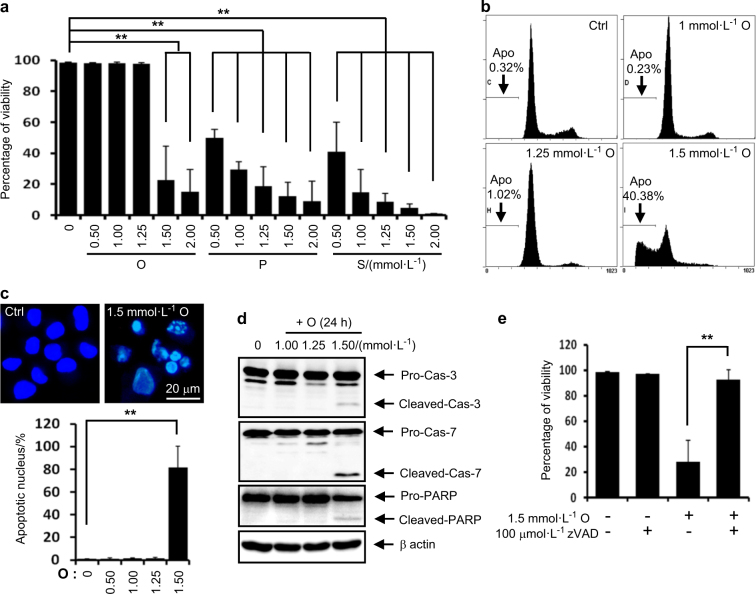
FFAs exert lipotoxicity in rat articular chondrocytes. **a** Viability assay. Palmitate (P) or stearate (S) at 0.5–2 mmol·L^-1^ and oleate (O) at 1.5–2.0 mmol·L^-1^ significantly reduced cell viability (*n* = 4). **P < 0.01 vs. vehicle according to Scheffe’s test. **b** Representative histograms showing cell cycle progression and the induction of apoptosis (Apo, the percentage of the population undergoing apoptosis). Oleate at 1.5 mmol·L^-1^ markedly increased the number apoptotic cells. **c** Representative Hoechst staining. The quantification of staining demonstrates that 1.5 mmol·L^-1^ oleate significantly increased the number of cells with condensed apoptotic nuclei. **P < 0.01 vs. vehicle according to Scheffe’s test. Scale bar, 20 μm. **d** Representative western blots showing that 1.5 mmol·L^-1^ oleate significantly induced the activation of apoptosis-related factors (*n* = 4). **e** Viability assay showing that zVAD-fmk significantly prevented 1.5 mmol·L^-1^ oleate-induced cell death (*n* = 4). **P < 0.01 according to Scheffe’s test

Oleate at the usual clinical range dose not exert lipotoxicity, while oleate at a high pathological range exerts lipotoxicity in rat articular chondrocytes through apoptosis. We next examined whether FFAs exert lipotoxicity in rat articular chondrocytes. Although several studies have suggested that there is an increased FFA concentration in OA synovial fluid,^13,17^ no documented studies have reported the FFA levels in the synovial fluid of OA subjects. Although FFAs have been extensively used at concentrations of 50–750 μmol·L^-1^ in experimental studies;^18^ the FFA levels in plasma are usually only 0.2–2 mmol·L^-1^.^19,20^ Thus, in the present study, we screened the effect of FFAs at 0.1–2 mmol·L^-1^ on rat articular chondrocyte viability. We observed that palmitate or stearate at 0.5–2 mmol·L^-1^ reduced the viability of rat articular chondrocytes. While oleate at 0.5–1.25 mmol·L^-1^ did not reduce cell viability, oleate at 1.5–2.0 mmol·L^-1^ significantly reduced the viability of rat articular chondrocytes (Fig. 2a). Although most previous reports using oleate under 1.0 mmol·L^-1^ found that oleate did not exert lipotoxicity, our findings suggest that oleate at high pathological ranges exerts lipotoxicity in articular chondrocytes. We next examined the underlying mechanism by which toxic concentrations of oleate exert lipotoxicity. Quantification of DNA hypoploidy by flow cytometry showed that subdiploid apoptotic cells were accumulated (Fig. 2b) and evaluation of nuclear morphology demonstrated that nuclear condensation and fragmentation were increased (Fig. 2c). Furthermore, western blot assays showed that 1.5 mmol·L^-1^ oleate produced caspase-3 and -7, and PARP cleavage products (Fig. 2d) and a pancaspase inhibitor zVAD-fmk completely reversed the oleate-induced cytotoxicity (Fig. 2e). These data indicate that toxic concentrations of oleate exerted lipotoxicity in articular chondrocytes through apoptosis (Fig. 2b–e).

The maintenance of accumulated LD is associated with the resistance of articular chondrocytes to lipotoxicity.

**Fig. 3:**
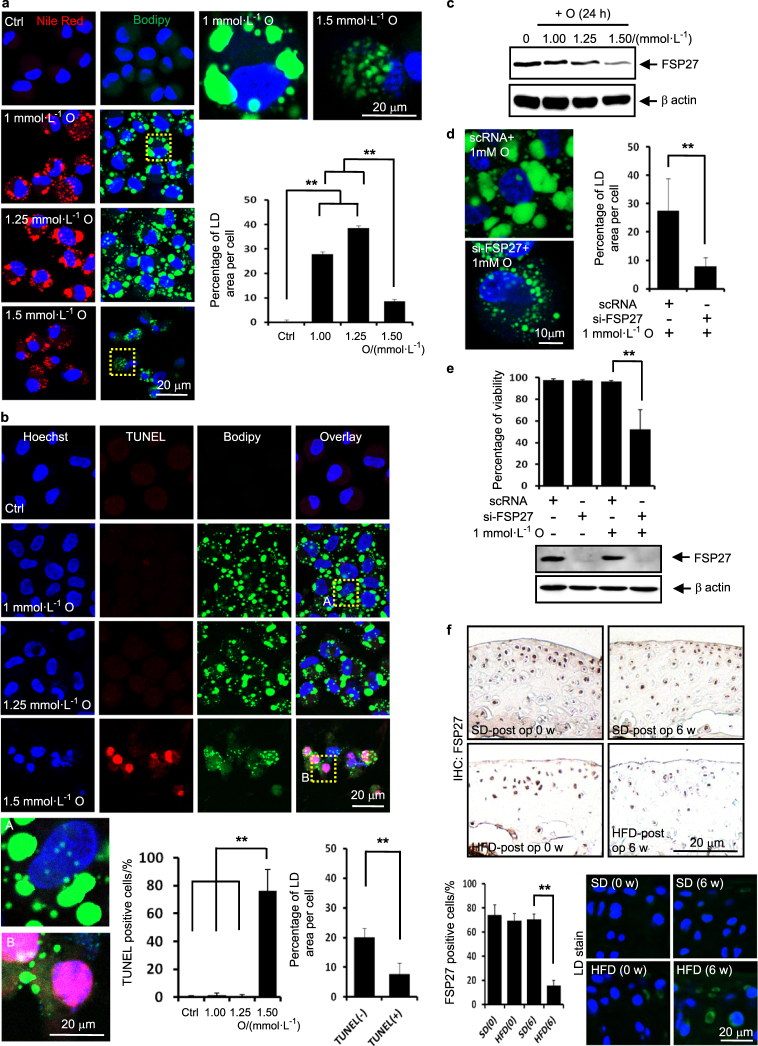
The maintenance of accumulated LD is associated with the resistance of articular chondrocytes to lipotoxicity. **a** Representative Nile red and BODIPY staining. Oleate at 1–1.25 mmol·L^-1^ induced giant LD. The quantification of the BODIPY staining showed that 1 and 1.25 mmol·L^-1^ oleate significantly increased the total LD volume (*n* = 4). **P < 0.01 vs. control according to Scheffe’s test. Compared to the cells treated with 1 and 1.25 mmol·L^-1^ oleate, the LD size was much smaller in cells treated with 1.5 mmol·L^-1^ oleate. The quantification of the BODIPY staining showed that 1.5 mmol·L^-1^ oleate significantly reduced the total LD volume (*n* = 4). **P < 0.01 vs. 1 and 1.25 mmol·L^-1^ oleate treatment according to Scheffe’s test. Scale bars, 20 μm. **b** Representative TUNEL, Hoechst and BODIPY triple-labelling showing that more TUNEL-positive cells are observed in cells treated with toxic concentrations of oleate (1.5 mmol·L^-1^) compared to control cells or cells treated with nontoxic concentrations of oleate (1 and 1.25 mmol·L^-1^). The quantification of TUNEL staining showed that 1.5 mmol·L^-1^ oleate significantly increased the population of TUNEL-positive cells. **P < 0.01 vs. control and 1 and 1.25 mmol·L^-1^ oleate treatment according to Scheffe’s test. The size of the LDs decreased in TUNEL-positive cells compared to TUNEL-negative cells. The quantification of BODIPY staining showed that the total LD volume was significantly decreased in TUNEL-positive cells. **P < 0.01 vs. TUNEL-negative cells according to Scheffe’s test. Scale bars, 20 μm. **c** Representative western blots showing that 1.5 mmol·L^-1^ oleate decreased the expression level of FSP27 protein (*n* = 4). **d** The knockdown of FSP27 reduced the total LD volume. The quantification shows that the knockdown of FSP27 significantly decreased the total LD volume. **P < 0.01 vs. scRNA control according to Scheffe’s test. Scale bar, 10 μm. **e** Viability assay showing that FSP27 depletion significantly sensitised chondrocytes to lipotoxicity (*n* = 4). **P < 0.01 vs. scRNA according to Scheffe’s test. **f** Immunohistochemistry on cartilages obtained from ACLT model 6 weeks after surgery. The FSP27-positive cells were quantified (*n* = 4). The population of FSP27-positive articular chondrocytes was significantly smaller in mice fed an HFD than in mice fed an SD. **P < 0.01 vs. mice fed an SD according to Scheffe’s test. BODIPY and Hoechst dual labelling showing that LDs are accumulated in mice fed an HFD. Scale bar, 20 μm

By investigating the differential effect of oleate at toxic and nontoxic concentrations of oleate, we further seek the defence mechanism in articular chondrocytes to resist lipotoxicity. Confocal microscopy demonstrated that giant LDs formed in chondrocytes treated with nontoxic concentrations of oleate. Importantly, toxic concentrations of oleate reduced the size of LDs and the total LD volume (Fig. 3a). Toxic concentration of oleate significantly increased the population of TUNEL-positive cells and giant LDs were not observed in TUNEL-positive cells. The total LD volume was significantly reduced in TUNEL-positive cells undergoing apoptosis compared with that of TUNEL-negative cells (Fig. 3b). We next examined whether knockdown of FSP27, which is associated with the surface of intracellular LD, involves in the resistance of articular chondrocytes to lipotoxicity. Western blot assays showed that toxic concentrations of oleate decreased the expression level of FSP27 (Fig. 3c) and the knockdown of FSP27 markedly reduced the total LD volume in articular chondrocytes (Fig. 3d). Notably, 1 mmol·L^-1^ oleate substantially induced cell death in FSP27-depleted chondrocytes, indicating that FSP27 depletion sensitises chondrocytes to lipotoxicity (Fig. 3e). These in vitro data indicate that LD accumulation through FSP27 is associated with the resistance of articular chondrocytes to oleate-induced lipotoxicity. Immunohistochemistry and BODIPY labelling on cartilages obtained from ACLT model 6 weeks after surgery show that the population of FSP27-positive articular chondrocytes was significantly smaller in mice fed an HFD than in mice fed an SD and LDs are accumulated in mice fed an HFD (Fig. 3f). These data suggest that FSP27 confers articular chondrocytes the ability to resist lipotoxicity through maintaining LD accumulation.

### PKCK2 confers articular chondrocytes the ability to resist lipotoxicity through maintaining LD accumulation

**Fig. 4:**
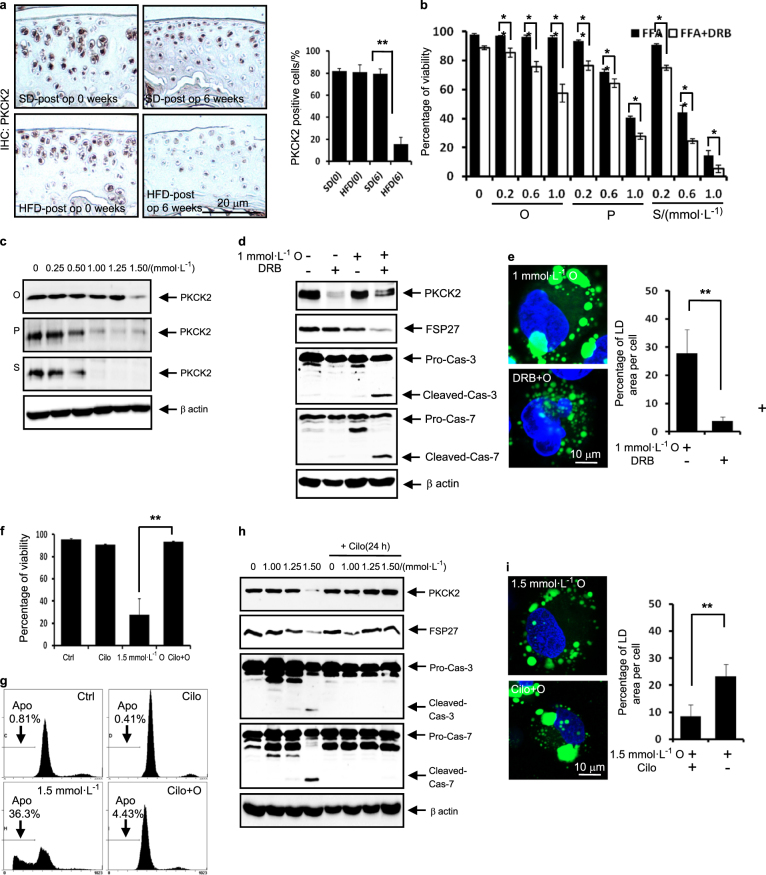
PKCK2 confers articular chondrocytes the ability to resist lipotoxicity through maintaining LD accumulation. **a** Immunohistochemistry staining of the cartilage obtained from ACLT model shows that the population of PKCK2-positive articular chondrocytes 6 weeks after surgery was significantly smaller in mice fed an HFD than in mice fed an SD. The PKCK2-positive cells were quantified (*n* = 4). **P < 0.01 vs. mice fed an SD according to Scheffe’s test. Scale bar, 20 μm. **b** Viability assay showing that FFAs sensitised chondrocytes to 100 μmol·L^-1^ DRB-induced death (*n* = 4). *P < 0.05 and **P < 0.01 according to Scheffe’s test. **c** Representative western blots showing that FFAs at toxic concentrations reduced the expression level of PKCK2 protein (*n* = 4). **d** Representative western blots showing that 1 mmol·L^-1^ oleate with 100 μmol·L^-1^ DRB activated caspase-3 and -7 and decreased the levels of FSP27 protein (*n* = 4). **e** Representative confocal microscopy images showing that co-treatment with DRB reversed oleate-induced LD enlargement. The quantification shows that the total LD volume was significantly decreased by co-treatment with DRB (*n* = 4). **P < 0.01 vs. 1 mmol·L^-1^ oleate treatment according to Scheffe’s test. Scale bar, 10 μm. **f** Viability assay showing that cilostazol (Cilo) pre-treatment prevented 1.5 mmol·L^-1^ oleate-induced cell death (*n* = 4). **P < 0.01 vs. oleate alone treatment according to Scheffe’s test. **g** Representative histograms showing that 30 μmol·L^-1^ cilostazol prevented 1.5 mmol·L^-1^ oleate-induced apoptosis (*n* = 4). **h** Representative western blots showing that 30 μmol·L^-1^ cilostazol prevented the 1.5 mmol·L^-1^ oleate-induced downregulation of FSP27 and activation of caspase-3 and -7 (*n* = 4). **i** Representative confocal microscopy images showing that cilostazol prevented oleate-induced LD fragmentation (*n* = 4). The quantification shows that the total LD volume was significantly increased by cilostazol. **P < 0.01 vs. 1.5 mmol·L^-1^ oleate alone treatment according to Scheffe’s test. Scale bar, 10 μm

PKCK2 is an important signal participating in articular chondrocyte death.^21,22^ Elevated FFAs should act synergistically with destructive stimuli in the pathogenesis of OA. A previous study reported that FFAs augment chondrocyte death through IL-1-β.^16^ We here examined whether FFAs act with PKCK2 in the pathogenesis of OA. Immunohistochemistry analysis of ACLT model demonstrated that the population of PKCK2-positive cells was significantly lower in mice fed an HFD than in mice fed an SD (Fig. 4a). These data suggest that PKCK2 prohibits dietary fat-induced lipotoxicity in articular chondrocytes. Thus, we further examined the detailed mechanism underlying the prohibition of lipotoxicity in vitro. Our viability assay revealed that 5,6-dichlorobenzimidazol riboside (DRB), a PKCK2 inhibitor, sensitised chondrocytes to cell death induced by all types of FFA tested (Fig. 4b). We further examined whether FFAs downregulate PKCK2. Noticeably, toxic concentrations of all FFAs reduced the expression level of PKCK2 protein. And we observed that oleate at 1.5 mmol·L^-1^, but not at 1–1.25 mmol·L^-1^, markedly reduced the expression level of the PKCK2 protein (Fig. 4c). We next examined whether PKCK2 inhibition sensitises chondrocytes to lipotoxicity. Western blot assays demonstrated that 1 mmol·L^-1^ oleate in conjunction with DRB induced the activation of pro-apoptotic caspase-3 and -7 in articular chondrocytes. While 1 mmol·L^-1^ oleate did not alter the FSP27 protein expression level, 1 mmol·L^-1^ oleate plus 100 μmol·L^-1^ DRB decreased the FSP27 protein level (Fig. 4d). We also observed that co-treatment with DRB reduced 1 mmol·L^-1^ oleate-induced LD accumulation (Fig. 4e). These findings suggest that PKCK2 inhibition prohibits oleate-caused LD enlargement, resulting in the induction of apoptosis. Because cilostazol prevents the reduction of PKCK2 activity in rat articular chondrocytes,^22^ we next examined whether the upregulation of PKCK2 by cilostazol would protect articular chondrocytes from oleate-induced lipoapoptosis. We observed that cilostazol pre-treatment prevented oleate-induced lipoapoptosis (Fig. 4f–h). Notably, cilostazol reversed the oleate-induced decrease in the FSP27 protein expression level (Fig. 4h). Confocal microscopy further showed that the prevention of chondrocyte apoptosis by cilostazol was associated with the reduction of the total LD volume (Fig. 4i). These findings indicate that PKCK2 confers articular chondrocytes the ability to resist lipotoxicity through maintaining LD accumulation.

### STAMP2 confers articular chondrocytes the ability to resist lipotoxicity through maintaining LD accumulation

**Fig. 5:**
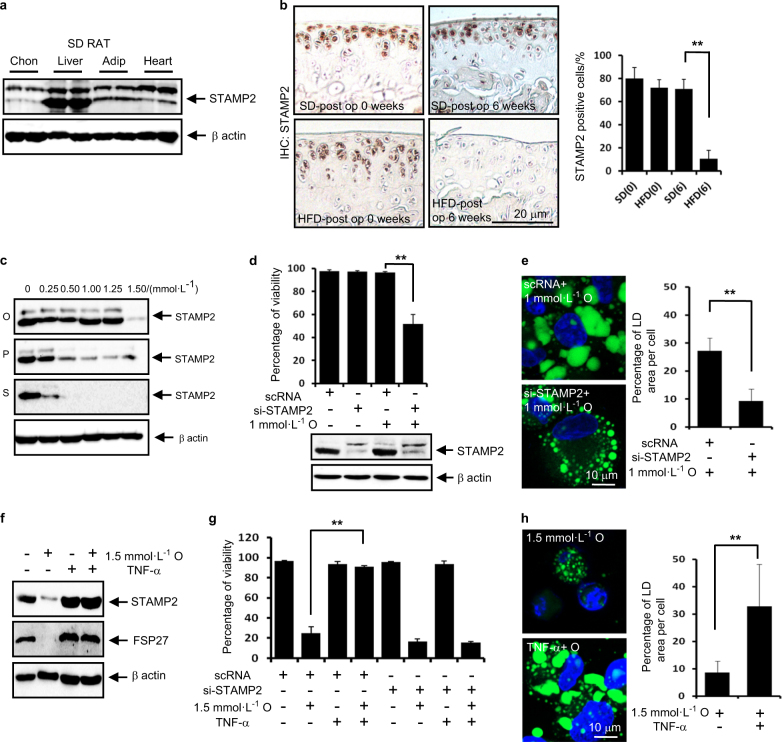
STAMP2 confers articular chondrocytes the ability to resist lipotoxicity through maintaining LD accumulation. **a** Representative western blots showing that STAMP2 was substantially expressed in rat articular chondrocytes (Chon) (*n* = 4). Liver, adipose (Adip) and heart tissue were used as positive controls. **b** Immunohistochemistry staining of the cartilage obtained from ACLT model shows that the population of STAMP2-positive articular chondrocytes 6 weeks after surgery was significantly smaller in mice fed an HFD than in mice fed an SD. The STAMP2-positive cells were quantified (*n* = 4). **P < 0.01 vs. mice fed an SD according to Scheffe’s test. Scale bar, 20 μm. **c** Representative western blots showing that oleate, palmitate or stearate at toxic concentrations reduced the expression level of STAMP2 (*n* = 4). **d** Viability assay showing that STAMP2-depletion significantly sensitised chondrocytes to lipotoxicity (*n* = 4). **P < 0.01 vs. scRNA according to Scheffe’s test. **e** The knockdown of STAMP2 reduced the total LD volume. The quantification shows that the knockdown of STAMP2 significantly decreased the total LD volume. **P < 0.01 vs. scRNA control according to Scheffe’s test. Scale bar, 10 μm. **f** Representative western blots showing that 25 ng·mL^-1^ TNF-α reversed 1.5 mmol·L^-1^ oleate-induced downregulation of STAMP2 protein (*n* = 4). **g** Viability assay showing that 25 ng·mL^-1^ TNF-α prevented 1.5 mmol·L^-1^ oleate-induced cell death (*n* = 4), which was not observed in STAMP2 (ST2)-depleted cells. **P < 0.01 vs. 1.5 mmol·L^-1^ oleate alone treatment. **h** TNF-α reversed 1.5 mmol·L^-1^ oleate-induced reduction of LD size and total LD volume. **P < 0.01 vs. 1.5 mmol·L^-1^ oleate alone treatment. Scale bar, 10 μm

STAMP2 plays a pivotal role in lipid homoeostasis.^23–25^ In the present study, we observed for the first time that STAMP2 is substantially expressed in rat articular chondrocytes (Fig. 5a). Thus, we next examined whether STAMP2 is involved in the pathophysiology of HFD-associated OA. Immunohistochemistry analysis of ACLT model demonstrated that the population of STAMP2-positive cells was significantly lower in mice fed an HFD than in mice fed an SD (Fig. 5b). These data suggest that STAMP2 prohibits dietary fat-induced lipotoxicity in articular chondrocytes. Thus, we further examined the detailed mechanism underlying the prohibition of lipotoxicity in vitro. We observed that toxic concentrations of all FFAs tested markedly reduced the expression level of STAMP2 (Fig. 5c). We also examined the effect of knockdown of STAMP2 on oleate-induced lipotoxicity. Notably, 1 mmol·L^-1^ oleate substantially induced cell death in STAMP2-depleted chondrocytes, indicating that STAMP2 depletion sensitises chondrocytes to lipotoxicity (Fig. 5d). In addition, we observed that the knockdown of STAMP2 markedly reduced the total LD volume (Fig. 5e). We next examined the effect of TNF-α, which has been reported to upregulate STAMP2,^24^ on oleate-induced lipotoxicity. TNF-α completely reversed the 1.5 mmol·L^-1^ oleate-induced downregulation of the expression level of FSP27 as well as STAMP2 protein (Fig. 5f). A viability assay further showed that TNF-α completely reversed 1.5 mmol·L^-1^ oleate-induced cell death, which was not observed in STAMP2-depleted cells (Fig. 5g). Furthermore, TNF-α markedly reversed the 1.5 mmol·L^-1^ oleate-induced reduction in total LD volume (Fig. 5h). These findings suggest that STAMP2 confers articular chondrocytes resistance to lipotoxicity through maintaining LD accumulation.

### The PKCK2/STAMP2/FSP27 axis confers articular chondrocytes resistance to lipotoxicity

**Fig. 6:**
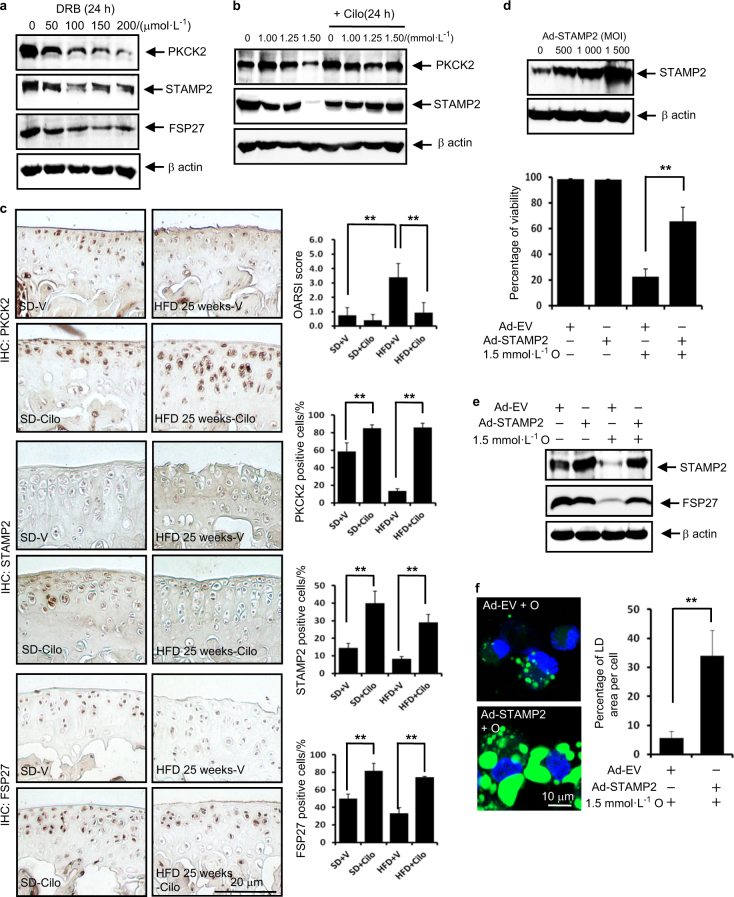
The PKCK2/STAMP2/FSP27 axis confers articular chondrocytes resistance to lipotoxicity. **a** Representative western blots showing that DRB decreased the expression level of STAMP2 and FSP27 (*n* = 4). **b** Representative western blots showing that 30 μmol·L^-1^ cilostazol reversed the 1.5 mmol·L^-1^ oleate-induced downregulation of STAMP2 (*n* = 4). **c** Representative immunohistochemistry on cartilages obtained from OA model without surgery. Cilostazol markedly prevented the HFD-induced cartilage destruction. OARSI scoring demonstrated that cilostazol significantly reduced the degeneration of cartilage. **P < 0.01 according to Scheffe’s test. Cilostazol significantly increased the populations of PKCK2, STAMP2 or FSP27-positive cells irrespective of diet type (*n* = 4). **P < 0.01 vs. vehicle (V) administered experimental control mice according to Scheffe’s test. Scale bar, 20 μm. **d** Representative western blots showing that STAMP2 was efficiently overexpressed by Ad-STAMP2 in articular chondrocytes. Viability assay showing that overexpressed STAMP2 significantly prevented oleate-induced cell death. **P < 0.01 vs. 1.5 mmol·L^-1^ oleate alone plus empty vector (Ad-EV) treatment according to Scheffe’s test. **e** Representative western blots showing that overexpressed STAMP2 (1 000 MOI) reversed the 1.5 mmol·L^-1^ oleate-induced decrease in FSP27 protein expression levels (*n* = 4). **f** The overexpression of STAMP2 (1 000 MOI) reversed the 1.5 mmol·L^-1^ oleate-induced reduction in the total LD volume. **P < 0.01 vs. empty vector according to Scheffe’s test. Scale bar, 10 μm

We next examined the hierarchical regulation of the resistance to lipotoxicity. We first observed that PKCK2 inhibition decreased the expression levels of FSP27 and STAMP2 in vitro (Fig. 6a). Furthermore, the PKCK2 augmenter cilostazol completely reversed the downregulation of STAMP2 induced by oleate in vitro (Fig. 6b). In conjunction with the data shown in Fig. 4h, these findings suggest that PKCK2 functions upstream of STAMP2 and FSP27. Thus, we next examined the effect of cilostazol on HFD-induced cartilage destruction using the OA model without surgery. We observed that cilostazol administration markedly prevented the HFD-induced cartilage destruction. Additionally, OARSI scoring demonstrated that cilostazol significantly reduced the degeneration of cartilage (Fig. 6c). We further examined the effect of cilostazol on the populations of PKCK2-, STAMP2- or FSP27-positive cells. Noticeably, cilostazol significantly increased the population of STAMP2- or FSP27-positive chondrocytes as well as PKCK2-positive cells irrespective of diet type (Fig. 6c). We next examined the effect of STAMP2 overexpression on oleate-induced lipotoxicity. Overexpression of STAMP2 not only significantly prevented 1.5 mmol·L^-1^ oleate-induced cell death (Fig. 6d), but also reversed the oleate-induced decrease in the FSP27 protein expression level (Fig. 6e). Furthermore, overexpression of STAMP2 reversed the 1.5 mmol·L^-1^ oleate-induced reduction in the total LD volume (Fig. 6f). The data in Fig. 6d–f in conjunction with the data shown in Fig. 5f indicate that STAMP2 functions upstream of FSP27. We next examined whether the staining of these proteins is reduced because the cells expressing them have already undergone apoptosis. The decreased populations of PKCK2-, STAMP2- or FSP27-positive cells were highly correlated with the increase of apoptosis level two in vivo OA models (Supplementary information 1a). However, the percentage of TUNEL-positive cell is merely within ~1/6–1/2 that of the percentage of PKCK2-, STAMP2- or FSP27-negative cells. In addition, we observed that PKCK2, STAMP2 and FSP27 proteins are downregulated at earlier time points than the time point of caspase activation (Supplementary information 1b). These data suggest that downregulation of PKCK2, STAMP2 and FSP27 proteins precedes the occurrence of apoptosis.

### LD accumulation rescues articular chondrocytes co-incubated with palmitate and oleate in vitro and articular chondrocytes of mice fed an HFD in vivo

**Fig. 7:**
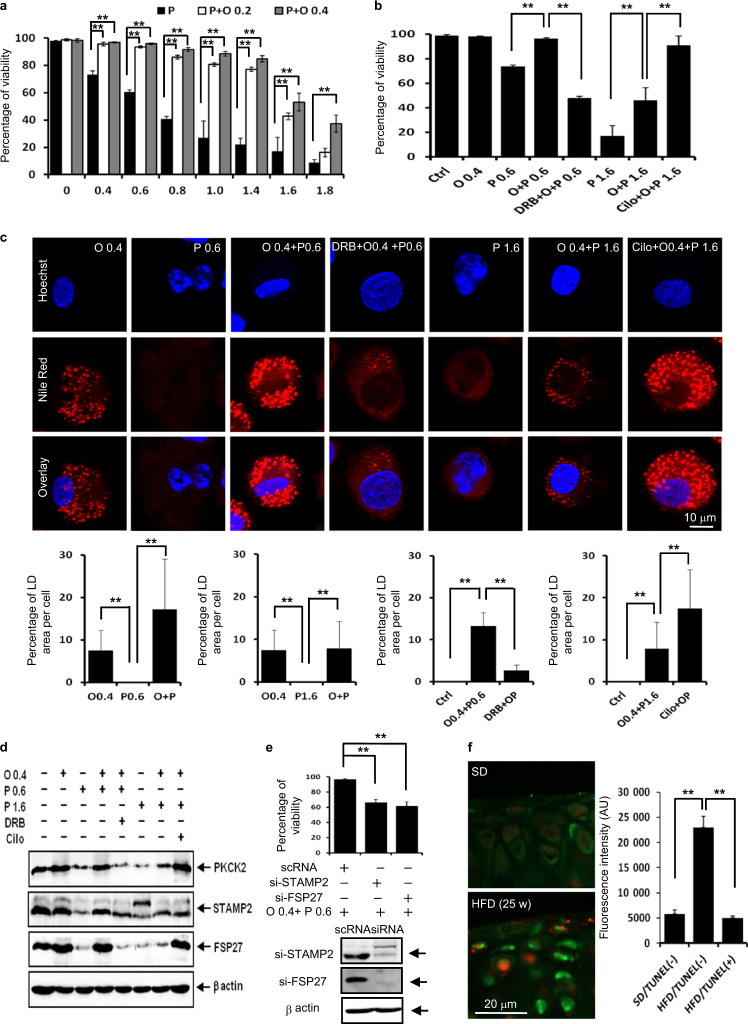
LD accumulation rescues articular chondrocytes co-incubated with palmitate and oleate in vitro and articular chondrocytes of mice fed an HFD in vivo. **a** Viability assay showing that 0.2 or 0.4 mmol·L^-1^ oleate supplementation suppressed palmitate-induced lipotoxicity (*n* = 4). **P < 0.01 vs. experimental control (palmitate alone treatment) according to Scheffe’s test. **b** Viability assay. Oleate at 0.4 mmol·L^-1^ significantly reversed 0.6 and 1.6 mmol·L^-1^ palmitate-induced lipotoxicity. PKCK2 inhibition by DRB and PKCK2 augmentation by cilostazol showed reciprocal influences on lipotoxicity (*n* = 4). **P < 0.01 vs. experimental control according to Scheffe’s test. **c** Representative confocal microscopy images. Oleate supplementation induced LD accumulation in articular chondrocytes treated with 0.6 or 1.6 mmol·L^-1^ palmitate. While DRB reduced LD accumulation in cells treated with 0.4 mmol·L^-1^ oleate plus 0.6 mmol·L^-1^ palmitate, cilostazol augmented LD accumulation in cells treated with 0.4 mmol·L^-1^ oleate plus 1.6 mmol·L^-1^ palmitate. LD accumulation was associated with resistance to the lipotoxicity in chondrocytes co-treated with oelate and palimate (*n* = 4). **P < 0.01 vs. control or experimental control according to Scheffe’s test. Scale bar, 10 μm. **d** Representative western blots showing that the increase in the LD accumulation in chondrocytes co-treated with 0.4 mmol·L^-1^ oleate and 0.6 mmol·L^-1^ palmitate or 0.4 mmol·L^-1^ oleate and 1.6 mmol·L^-1^ palmitate was correlated with the increase in the expression level of PKCK2, STAMP2 and FSP27 (*n* = 4). **e** Viability assay showing that siSTAMP2 and siFSP27 significantly decreased the viability of chondrocytes co-treated with 0.4 mmol·L^-1^ oleate and 0.6 mmol·L^-1^ palmitate (*n* = 4). **P < 0.01 vs. scRNA-administered experimental control according to Scheffe’s test. **f** Representative TUNEL and BODIPY double-labelling on cartilages obtained from OA model without surgery. The BODIPY fluorescence intensity in articular chondrocytes of mice fed an SD is lower compared to TUNEL-negative cells of mice fed an HFD. The quantification of BODIPY staining showed that the total LD volume was significantly increased in TUNEL-negative cells of mice fed an HFD (*n* = 4). **P < 0.01 vs. articular chondrocytes of mice fed an SD according to Scheffe’s test. The BODIPY fluorescence intensity in TUNEL-positive cells of mice fed HFD is lower compared to TUNEL-negative cells. The quantification of BODIPY staining showed that the total LD volume was significantly reduced in TUNEL-positive cells (*n* = 4). **P < 0.01 vs. TUNEL-negative cells according to Scheffe’s test. Scale bar, 20 μm

Under physiological conditions, the FFA pool contains different saturated and unsaturated species that influence each other. Palmitate and oleate are two of the most common fatty acids in articular chondrocytes.^26^ We observed that 0.2 and 0.4 mmol·L^-1^ oleate supplementation markedly suppressed 0.4–1.8 mmol·L^-1^ palmitate-induced lipotoxicity (Fig. 7a). For further studies on LD accumulation, we chose the following two combination treatments: 0.4 mmol·L^-1^ oleate with 0.6 mmol·L^-1^ palmitate and 0.4 mmol·L^-1^ oleate with 1.6 mmol·L^-1^ palmitate. In these two combination treatments, oleate supplementation significantly reversed palmitate-induced lipotoxicity, and PKCK2 inhibition and augmentation showed reciprocal influences on lipotoxicity (Fig. 7b). In these combination treatments, oleate supplementation induced LD accumulation in articular chondrocytes treated with 0.6 mmol·L^-1^ or 1.6 mmol·L^-1^ palmitate. While DRB significantly reduced the LD accumulation in cells treated with 0.4 mmol·L^-1^ oleate plus 0.6 mmol·L^-1^ palmitate, cilostazol augmented the LD accumulation in cells treated with 0.4 mmol·L^-1^ oleate plus 1.6 mmol·L^-1^ palmitate (Fig. 7c). These findings support the notion that LD accumulation also prevents lipotoxicity in articular chondrocytes co-incubated with palmitate and oleate. We further observed that the increase in LD accumulation in chondrocytes co-treated with oleate and palmitate also correlated with the increase in the expression level of PKCK2, STAMP2 and FSP27 (Fig. 7d). Moreover, siSTAMP2 and siFSP27 significantly decreased the viability of chondrocytes co-treated with 0.4 mmol·L^-1^ oleate and 0.6 mmol·L^-1^ palmitate (Fig. 7e). The data in Figs. 6 and 7 indicate that the PKCK2/STAMP2/FSP27 axis confers articular chondrocytes resistance to FFA-induced lipotoxicity. We further examined whether LD accumulation is associated with the resistance to lipotoxicity in vivo. Noticeably, the total LD volume was significantly reduced in TUNEL-positive cells compared with that of TUNEL-negative cells in cartilages obtained from OA model without surgery (Fig. 7f).

### An increase in cytosolic FFAs is correlated with the exertion of lipotoxicity in articular chondrocytes

**Fig. 8:**
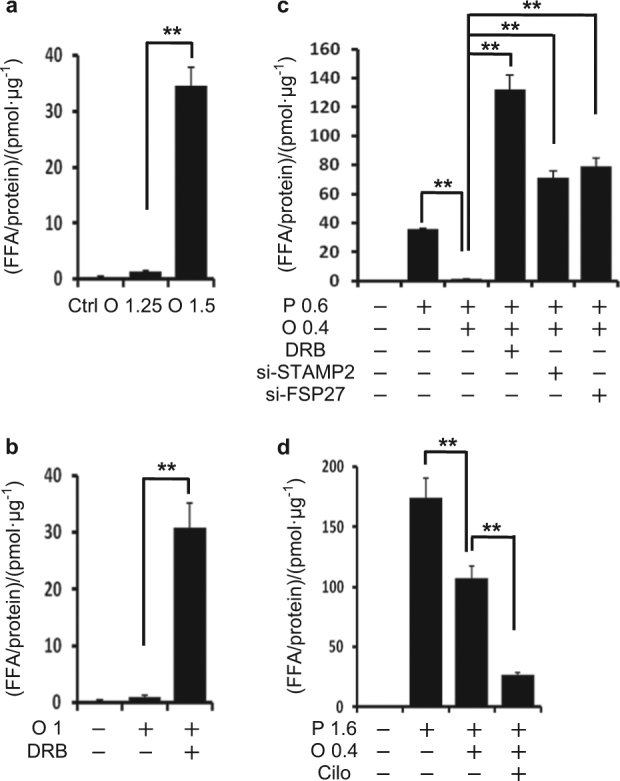
The total cytosolic FFA content is higher in articular chondrocytes that succumb to FFA-induced lipotoxicity than in articular chondrocytes that survive lipotoxicity. **a** The FFA level in the cytosol of articular chondrocytes treated with 1.25 or 1.5 mmol·L^-1^ oleate. 1.5 mmol·L^-1^ oleate significantly increased FFA level in the cytosol compared to experimental control (1 mmol·L^-1^ oleate). **P < 0.01 vs. experimental control according to Scheffe’s test. **b** The FFA level in the cytosol of articular chondrocytes treated with 1.0 mmol·L^-1^ oleate with or without DRB. DRB significantly increased the FFA level in the cytosol. **P < 0.01 vs. experimental control (oleate alone) according to Scheffe’s test. **c** The FFA level in the cytosol of articular chondrocytes treated with 0.6 mmol·L^-1^ palmitate with or without 0.4 mmol·L^-1^ oleate. Oleate supplementation significantly decreased the FFA level in the cytosol of articular chondrocytes treated with 0.6 mmol·L^-1^ palmitate. DRB, siSTAMP2 and siFSP27 significantly reversed this reduction of FFA level by oleate supplementation (*n* = 4). **P < 0.01 vs. experimental control according to Scheffe’s test. **d** The FFA level in the cytosol of articular chondrocytes treated with 1.6 mmol·L^-1^ palmitate with or without 0.4 mmol·L^-1^ oleate. Oleate supplementation significantly decreased the FFA level in cells treated with 1.6 mmol·L^-1^ palmitate. Cilostazol significantly decreased the FFA level in the cytosol of cells co-treated with 1.6 mmol·L^-1^ palmitate and 0.4 mmol·L^-1^ oleate (*n* = 4). **P < 0.01 vs. experimental control according to Scheffe’s test

We next examined whether cytosolic FFAs, which are not incorporated in LDs, are increased in articular chondrocytes that succumb to lipotoxicity. We measured the total cytosolic FFA content in articular chondrocytes after various combination treatments. Notably, we observed that the concentrations of cellular FFAs were significantly higher in articular chondrocytes that succumbed to lipotoxicity compared with those in articular chondrocytes that survived FFA-induced lipotoxicity (Fig. 8a–d). This phenotype was observed not only in articular chondrocytes treated with oleate alone (Fig. 8a, b), but also in cells co-treated with palmitate and oleate (Fig. 8c, d). These findings indicate that the exertion of lipotoxicity by FFAs seems to depend on the increased concentration of cellular FFAs freed from LDs, supporting the conclusion that articular chondrocytes survive through the sequestration of FFAs in LDs.

## Discussion

The accumulation of excessive lipids in non-adipose tissues in hyperlipidaemic states can lead to lipotoxicity, cell dysfunction and/or cell death. Although it is clear that lipids naturally accumulate in chondrocytes,^9,13^ it was recently reported that the accumulation of FFAs contributes to chondrocyte dysfunction related to the pathogenesis of OA. We in the present study revealed that HFD accelerates the onset of OA and that which FFAs exert lipotoxicity in articular chondrocytes through apoptosis. We further revealed the mechanism by which articular chondrocytes survive under the effects of FFA accumulation.

Cells have developed the capacity to store fatty acids as neutral lipids within LDs. While adipocytes store fatty acids as triacylglycerol to serve as a reservoir for energy that can be released when food is scare, cells in non-adipose tissues protect themselves from the detrimental effects originating from excess fatty acids by sequestering them within LDs.^27^ Triglyceride accumulation in non-adipose tissues occurs in the setting of mismatch between cellular lipid influx and lipid utilisation.^28^ It is well known that chondrocytes contain LDs in their cytoplasm.^11,12,29^

In different experimental systems, saturated and unsaturated FFAs differ significantly in their contributions to lipotoxicity. While lipotoxicity from the accumulation of long-chain FFAs is specific for saturated FFAs, co-supplementation with unsaturated FFAs rescues saturated FFA-induced lipoapoptosis.^30^ It is a well-known metabolic phenomenon that triglycerides accumulate in response to the increased cellular level of unsaturated FFAs. Not only exogenous oleate but endogenously generated unsaturated FFAs lead to triglyceride accumulation.^28^ The mechanism of cellular triglyceride accumulation by unsaturated FFAs has been demonstrated in several studies. One such study dissected the molecular role of cellular triglyceride accumulation by unsaturated FFAs. The study revealed that unsaturated FFAs rescue palmitate-induced apoptosis by channelling palmitate into triglyceride pools, which suggests that the accumulation of excess FFAs in triglyceride pools likely diverts these molecules from pathways leading to lipotoxicity and serves as a buffer against lipotoxicity.^28^

The present study is the first to demonstrate that triglyceride accumulation in response to oleate supplementation represents a defence against lipotoxicity in articular chondrocytes. Because articular chondrocytes are unable to utilise accumulated lipids for energy purposes,^31^ this accumulation of excess FFAs in triglyceride pools or LDs seems to divert FFAs from the pathway leading to lipotoxicity. While articular chondrocytes protect themselves from lipotoxicity by sequestering palmitate within LDs, lipotoxicity by palmitate likely occurs when either the cellular capacity for triglyceride storage is exceeded or LDs are destroyed, resulting in increased cellular palmitate. In addition, our observations offer a new perspective on the exhibition of lipotoxicity by unsaturated FFAs. We observed that oleate, at a concentration that degrades LDs and increases cellular FFAs, also exerted lipotoxicity, while supplementation with oleate at a concentration that leads to triglyceride accumulation was well tolerated. Based on these findings, the exhibition of lipotoxicity by FFAs in chondrocytes seems to depend on the increased concentration of FFAs freed from LDs, whether FFAs are saturated or not. Although the duration and extent of lipid overload, in general, may determine whether a cell is protective or damaged, oleate after a rather small changes in concentration (1.25–1.5 mmol·L^-1^) markedly reduced chondrocytes viability. Within this narrow range of oleate concentration, oleate storage capacity seems to be exceeded and triglyceride appears to be eventually hydrolysed, abruptly increasing toxic lipids to exert lipotoxicity.^32^ However, for understanding the molecular mechanism underlying this abrupt lipotoxicity exerted by oleate in chondrocytes, further future study is required. Because most previous studies have shown that co-supplementation with unsaturated FFAs rescues saturated FFA-induced lipoapoptosis, determining whether the exhibition of lipotoxicity by unsaturated FFAs freed from LDs is observable in other cellular contexts is an urgent challenging task for future research.

Arguably, the most intriguing finding of our study is the participation of PKCK2/STAMP2/FSP27-mediated LD accumulation in the defence of articular chondrocytes against lipotoxicity. PKCK2 participates in a series of complex cellular functions, including cell growth and proliferation, by catalysing the phosphorylation of many proteins.^33^ PKCK2 also participates in the regulation of apoptosis by phosphorylating some apoptosis-related factors.^33,34^ Previous studies have demonstrated that PKCK2 is involved in anti-apoptotic effects against nitric oxide (NO)-induced apoptosis in rat articular chondrocytes and that the downregulation of PKCK2 facilitates TNF-α-mediated chondrocyte death through apoptosis and autophagy.^21^ Cilostazol, 6-[4-(1-cyclohexyl-1*H*-tetrazol-5-yl) butoxy]-3,4-dihydro-2-(1*H*)-quinolinone, is a potent selective phosphodiesterase type III (PDE III) inhibitor and is mainly used for treating patients with peripheral arterial disease and intermittent claudication. Previous studies have shown that cilostazol prevents apoptosis through PKCK2.^22^ The present study is the first to demonstrate that PKCK2 helped protect articular chondrocytes against FFA-induced lipoapoptosis.

STAMP2 plays a pivotal role in lipid homoeostasis, and the dysregulation of STAMP2 is implicated in metabolic and inflammatory diseases.^23,24,35^ Nutritional status, such as fasting or (re)feeding, changes STAMP2 expression in multiple tissues, including the liver, adipose tissue and muscle.^25,36^ STAMP2 expression was reduced in the livers of non-alcoholic fatty liver disease (NAFLD) patients and in the hepatocytes of mice fed an HFD.^25^ On the other hand, the STAMP2 mRNA and protein levels are low in preadipocytes but dramatically increase during the adipocyte differentiation process.^36^ Previous studies have shown that STAMP2 is induced by several pro-inflammatory cytokines, including TNF-α,^37^ and acts as an anti-inflammatory factor. Thus, in the context of inflammation, STAMP2 seems to play an important role. Indeed, several reports suggest that STAMP2 has a role in the pathogenesis of arthritis, as STAMP2 expression in the synovia correlates with the progression of joint swelling in both murine models and arthritis patients.^38,39^ Moreover, STAMP2-knockout mice spontaneously develop destructive arthritis.^40^ In this study, we first demonstrated that STAMP2 helped protect articular chondrocytes against FFA-induced lipoapoptosis.

FSP27, also known as CIDEC, belongs to the cell death-inducing DNA fragmentation factor 45 (DFF45)-like effector (CIDE) family of proteins.^41^ The ectopic expression of FSP27 promotes apoptosis in mammalian cells. In addition, FSP27 plays crucial roles in lipid metabolism^42^ and is closely linked to the development of metabolic disorders, including obesity, diabetes and liver steatosis.^43^ FSP27 also contributes to unilocular LD formation. The knockdown of FSP27 expression in well-differentiated adipocytes results in the loss of unilocular LDs and causes the accumulation of small multilocular LDs.^44^ The induction of FSP27 gene expression that occurs during the normal adipogenic programme, which is concomitant with LD accumulation, does not result in increased cellular apoptosis. Moreover, the physical localisation of FSP27 at LDs can inhibit its pro-apoptotic action.^42^ In this study, we first demonstrated that FSP27 helped protect articular chondrocytes against FFA-induced lipoapoptosis through LD accumulation. Although the present study revealed that PKCK2/STAMP2/FSP27-mediated LD accumulation mediates the defence of articular chondrocytes against lipotoxicity, the expression level of these proteins is not upregulated, neither at sub-apoptosis inducing doses of FFAs nor at the time-points earlier than the induction of apoptosis. These data indicate that sustaining the expression level of these proteins is required to mediate the defence against lipotoxicity. More detailed molecular mechanism underlying these molecules-mediated defence remains to be determined.

Irrespective of the data obtained from our experimental system, it is not clear that the increased OA is entirely due to a direct effect of LDs in articular chondrocytes. The increased OA might be due to other effects such as inflammation which is upregulated after prolonged HFD. It is also not clear that in vivo effect of cilostazol was entirely originated through PKCK2/STAMP2/FSP27 pathway. Because cilostazol has diverse activities, the in vivo effect of cilostazol might be originated from other potential efficacies such anti-inflammatory effect. Further future study on these issues is urgently required.

In conclusion, we demonstrated that articular chondrocytes protect themselves from lipotoxicity by sequestering FFAs within LDs and that the lipotoxicity of FFAs seems to depend on the increased concentration of cellular FFAs freed from LDs, whether FFAs are saturated or not. Because PKCK2/STAMP2/FSP27-mediated sequestration of free fatty acids in lipid droplets rescues dietary fat-associated osteoarthritic chondrocytes, PKCK2/STAMP2/FSP27 must be considered for interventions against metabolic OA.

## Materials and methods

### Reagents

The following reagents were obtained commercially: goat polyclonal anti-human CKIIα and HRP-conjugated donkey anti-goat IgGs antibodies from Santa Cruz Biotechnology (Santa Cruz, CA, USA); rabbit polyclonal anti-human caspase-3 and -7 antibodies from Cell Signaling (Danvers, MA, USA); rabbit polyclonal anti-human STAMP2 antibody from Proteintech (Rosemont, IL, USA); mouse monoclonal anti-FSP27 antibody from Abcam (Cambridge, MA, USA); HRP-conjugated donkey anti-rabbit and sheep anti-mouse IgGs from Amersham Pharmacia Biotech (Piscataway, NJ, USA); Ketamine hydrochloride from Sanofi-Ceva (Düsseldorf, Germany); Rompun from Bayer (Leverkusen, Germany); Dulbecco’s modified Eagle’s medium (DMEM) foetal bovine serum (FBS) and Opti-MEM from Gibco BRL (Gaithersburg, MD, USA); TNF-α and ApopTag FITC In Situ Apoptosis Detection Kit from Millipore (Temecular, CA); mouse monoclonal anti-human actin antibody, Hoechst 33342, dimethylsulphoxide (DMSO), RNase A, proteinase K, protein inhibitor, propidium iodide (PI), phenylmethylsulfonyl fluoride (PMSF) and 5,6-dichlorobenzimidazol riboside (DRB), fatty acid-free bovine serum albumin (BSA), Oil red O, palmitate, oleate, stearic acid, 3,3′-diaminobenzidine (DAB), cilostazol and type II collagenase from Sigma (St. Louis, MO, USA); caspase inhibitor I (zVAD-fmk) from Calbiochem (San Diego, CA, USA); 5,5′,6,6′-tetrachloro-1,1′,3,3′-tetraethylbenzimidazol carbocyanine iodide (JC-1), Nile red and BODIPY 493/503 from Molecular Probes (Eugene, OR, USA); siPORT Amin from Ambion (Austin, TX, USA); SuperSignal WestPico enhanced chemiluminescence Western blotting detection reagent from Pierce (Rockford, IL, USA).

### Animals

All procedures for the animal study were approved by the Committee on Animal Investigations at Dong-A University (DIACUC-15-12). Seven-week-old male C57BL/6 mice were obtained from Samtako (Osan, Korea). The mice were maintained in a temperature-controlled room (22 °C) on a 12:12 h light-dark cycle and given free access to water and food. Mice were fed an SD (standard diet) or HFD for the indicated durations. The SD comprised 16% fats, 64% carbohydrates (10% sucrose), 20% proteins, for a total of 4 kcal per 1 g of diet. The HFD comprised 60% fats, 20% carbohydrates (0% sucrose) and 20% proteins, for a total of 5.33 kcal per 1 g of diet. All components were purchased from FeedLab (Guri, South Korea).

### Mice subjected to surgery for experimental OA

Sixty mice were used. After eating a normal chow diet for 1 week, half of the mice were fed an HFD, and the other half were fed an SD. Mice fed an HFD or an SD for 8 weeks were subjected to surgery for experimental OA. The mice were anaesthetised with ketamine hydrochloride (15 mg·kg^-1^) and rompun (3.45 mg·kg^-1^). The anterior cruciate ligament was transected with a micro-surgical knife under direct visualisation, and complete transection was confirmed by the presence of the anterior drawer. After anaesthesia was released, the mice demonstrated excellent mobility within 2 h after surgery. At 4, 6 and 8 weeks after surgery, ten mice from each diet group were used for histological evaluation.

### HFD-induced OA mouse model without surgery

Sixty eight mice were fed a normal chow diet for 1 week, followed by an HFD or an SD for 25 weeks. Ten and four mice from each diet group were used to histologically examine the effect of the HFD and to observe LD, respectively. Forty mice were used to examine the effect of cilostazol. Mice fed an SD or HFD for 15 weeks were orally administered cilostazol at the concentration of 30 mg·kg^-1^ per day for an additional 10 weeks. SD g/vehicle mice (*n* = 10) were fed an SD and received DMSO. SD + cilostazol mice (*n* = 10) were fed an SD and received cilostazol. HFD + vehicle mice (*n* = 10) were fed an HFD and received DMSO. HFD + cilostazol mice (*n* = 10) were fed an HFD and received cilostazol.

### Tissue preparation

The animals were killed by ether inhalation. The whole knee joints were removed by dissection, fixed in PBS (pH 7.4) containing 4% paraformaldehyde, decalcified in 12.5% EDTA. For histologic and immunohistochemical analysis, knee joints were embedded in paraffin blocks and five-micrometre microsections were prepared. For LD analysis, decalcified knee joints were cryoprotected in a 20% sucrose solution and 10 μm sections were made using a cryostat (Frigocut).

### Histologic and immunohistochemical analyses

Five-micrometre microsections were prepared and stained with haematoxylin (Sigma-Aldrich) and eosin (Sigma-Aldrich) (H&E) as well as Safranin O (Sigma-Aldrich) -fast green (Sigma-Aldrich). For immunohistochemcial analyses, tissue sections from four animals of each group were incubated in 1:70-diluted goat serum solution for 30 min at room temperature and then for 2 h with the 1:100-diluted primary antibody at room temperature. Next, the sections were incubated with a secondary antibody for 1 h at 37 °C and developed using the ABC (Vector) complex. Peroxidase was revealed by DAB and examined by light microscopy. The histological images were observed and analysed using an Aperio ScanScope® CS system. The total numbers of cells positive for PKCK2, FSP27 and STAMP2 in four fields per animal were counted by an observer blinded to the experiment, and the percentages of positive cells were calculated.

### Cell culture of articular chondrocytes

Rat articular chondrocytes for primary culture were isolated from knee joint cartilage slices by enzymatic digestion for 1 h with 0.2% type II collagenase (381 U·mg^-1^, Sigma-Aldrich) in DMEM. After the isolated cells were collected by brief centrifugation, they were resuspended in DMEM supplemented with 10% (v/v) FBS, 50 mg·ml^-1^ streptomycin and 50 U·ml^-1^ penicillin (Gibco). The cells were plated on culture dishes at a density of 5 × 10^4^ cells/cm^2^. The medium was replaced every 2 days, and the cells reached confluency after ~5 days in culture. In each experiment, the cells from three animals were pooled and analysed three times.

### Treatment with FFAs or combination treatment with other chemicals

The FFAs were dissolved in absolute ethanol at a concentration of 500 mmol·L^-1^ and diluted to their final concentrations with the appropriate concentration of 1% (w/v) FFA-free BSA. Controls were incubated with equal concentrations of FFA-free BSA containing ethanol. To examine the effect of several chemicals, the cells were pre-treated with 150 μg·ml^-1^ DRB or 30 μmol·L^-1^ cilostazol for 24 h or 25 ng·ml^-1^ TNF-α for 3 h before FFA treatment.

### siRNA transfection or combination treatment with FFAs

Rat STAMP2 siRNA (SMARTpool; L-105419-02-0050) and FSP27 siRNA (SMARTpool; L-105647-02-0050) were purchased from Thermo Scientific (Hudson, NH, USA). As a negative control, the same nucleotides were scrambled to form nongenomic combinations. Transfection of the siRNA was performed with the use of siPORT Amine and Opti-MEM medium. Cells grown to a confluency of 40%–50% in six-well plates were transfected with 100 nmol·L^-1^ of siRNA per well. The transfection mixture was added to each well, and the cells were incubated for 4 h. Then, 2 mL of growth medium was added, and the cells were incubated for another 20 h. After the siRNA transfection medium was removed, cells were treated with FFAs for 24 h.

### Recombinant adenoviral STAMP2 infection

Recombinant adenoviral STAMP2 was prepared as described previously.^25^ Cells (1 × 10^7^) were infected with recombinant adenoviral STAMP2 at multiplicities of infection (MOIs) of 500, 1 000 and 1 500.

### Western blot analysis

Cells (2 × 10^6^) were washed twice with ice-cold PBS. Cells were resuspended in lysis buffer [200 μL of ice-cold solubilizing buffer (300 mmol·L^-1^ NaCl, 50 mmol·L^-1^ Tris–Cl (pH 7.6), 0.5% Triton X-100, protease inhibitor cocktail)] and incubated at 4 °C for 30 min. The lysates were centrifuged at 14 000 r·min^-1^ for 20 min at 4 °C. The protein concentrations of the cell lysates were measured with the Bradford protein assay reagent (Bio-Rad). Then, 50 μg of proteins was loaded onto 15% SDS-PAGE. The separted proteins were were transferred to nitrocellulose membranes (Amersham Pharmacia Biotech, Piscataway, NJ, USA) and probed with each antibody. Immunostaining with the antibodies was carried out using the Super Signal West Pico enhanced chemiluminescence substrate and detected with LAS-3000PLUS.

### Cell viability assay

An automated trypan blue exclusion assay was undertaken. Total cells and trypan blue-stained (i.e., nonviable) cells were counted, and the percentage of nonviable cells was calculated using the Vi-Cell cell counter (Beckman Coulter, Miami, FL, USA).

### Nuclear morphology study for apoptosis

Cells were collected and then washed with PBS. They were fixed in 4% paraformaldehyde for 20 min at room temperature. The cells were washed with PBS twice, and stained in 4 μg·mL^-1^ Hoechst 33342 for 1 h at 37 °C. Stained cells were coated onto clean, lipid-free glass slides and mounted with a cover glass. The samples were observed and photographed under an epifluorescence microscope (Axiophot, Zeiss, Germany). The number of cells that showed condensed or fragmented nuclei was determined by a blinded observer from a random sampling of 250–300 cells per experiment.

### Quantification of DNA hypoploidy and cell cycle phase analysis by flow cytometry

Cells were washed twice with PBS, and fixed with cold 70% ethanol at 4 °C overnight. The fixed cells were pelleted and ethanol was removed by washing twice with PBS containing 1% bovine serum albumin (BSA). The cells were resuspended in 1 mL of PBS containing 11 Kunitz U·mL^-1^ RNase A, incubated at 4 °C for 30 min and washed once with BSA/PBS. Cells were resuspended in PI solution (50 μg·mL^-1^) and incubated at 37 °C for 30 min in dark. Cells were washed with PBS, the DNA content of 10 000 cells was used for the generation of simultaneous estimation of the cell cycle parameters and apoptosis using an Epics XL (Beckman Coulter, FL).

### Assay of mitochondrial membrane potential

Disruption of mitochondrial membrane potential (MMP) was measured using a specific fluorescent probe, JC-1, that was added directly to the cell culture medium (5 μg·mL^-1^ final concentration) and incubated for 15 min at 37 °C. Cells were stained with JC-1, and flow cytometry to measure MMP was performed (an Epics XL; Beckman Coulter). Data were acquired and analysed using EXPO32 ADC XL 4 colour software.

### TUNEL staining of cell suspensions

Cell suspensions were cytospun onto clean fat-free glass slides in a cytocentrifuge. After being fixed with 4% paraformaldehyde, the cells were incubated with terminal deoxynucleotidyl transferase (TdT) enzyme for 1 h at 37 °C, and antidigoxigenin-FITC was applied for 30 min at room temperature. Afterward, nuclei were counterstained with PI/Antifade counterstain. Fluorescent images were observed and analysed using a Zeiss LSM 510 laser-scanning confocal microscope. Positive cells were counted by a blinded observer from a random sampling of 250–300 cells per experiment.

### Staining of LDs, confocal microscopy and quantification

Cells cultured on a coverslip and cryocut sections were incubated with diluted Nile red or BODIPY 493/503. Some were double-labelled with TUNEL and BODIPY 493/503 and counterstained with Hoechst 33342. Fluorescent images were observed and analysed using a Zeiss LSM 510 laser-scanning confocal microscope (Göettingen, Germany). Twenty cells from each experiment or animal were observed, and the quantification of the fluorescent intensity of the confocal images was performed using ZEN Blue analysis software.

### Total cytosol FFA content measurement

The cells were collected after treatment with trypsin (0.2% trypsin, 0.02% EDTA and 0.2% glucose in PBS) and pelleted by centrifugation (200 × *g* for 5 min at 4 °C). The cell pellet was resuspended in ice-cold hypotonic lysis medium containing 20 mmol·L^-1^ Tris-HCl at pH 7.4 and 1 mmol·L^-1^ EDTA. The cells were homogenised with a Dounce homogeniser and then centrifuged (800 × *g* for 5 min at 4 °C). The post-nuclear supernatant fraction was ultra-centrifuged (800 × *g* for 5 min at 4 °C) using a Beckman table-top ultracentrifuge. The LD fraction with a distinct white band on the preparation was removed with a pipette, and the LD-free cytosol was used for the FFA measurement. The cytosol FFA levels were measured using a commercial free fatty acid quantification kit from Abcam (ab65341, Cambridge, UK).

### Statistics

Four independent experiments performed in triplicate were conducted in vitro. Immunohistochemical and BODIPY stainings on tissues obtained from four animals of each group were quantified. The results are expressed as the mean ± SD from four experiments. Shapiro–Wilk test was conducted to check normality of data and Levene’s test verified homogeneity of variances before one-way analysis of variance (ANOVA). ANOVA followed by Scheffe’s test was used for the analysis of differences within each treated conditions. To test the statistical significance of the difference in the onset of OA between HFD and SD groups, we first generated a two-by-three contingency table with HFD/SD represented as rows and 4/6/8 weeks represented as columns, and then Pearson’s *χ*^2^ test was conducted to analyse the association between diet and time. To test the significance of an individual variable, we next fit the data with a Poisson regression model where the onset of OA in ten mice constituted the response and both types of diet (HFD/SD) and the three different weeks (4/6/8) constituted the predictors. The likelihood ratio test was used for the dietary variable. For the comparison between the HFD and SD for 25 weeks without surgery, the *χ*^2^ test for the same probabilities of the two groups was conducted.

## Electronic supplementary material

Supplementary information
