# Supplementary material for: Dietary fat-associated osteoarthritic chondrocytes gain resistance to lipotoxicity through PKCK2/STAMP2/FSP27
Source: Bone Res. 2018 Jul 6;6:20. doi: 10.1038/s41413-018-0020-0 (PMC6033867; doi:10.1038/s41413-018-0020-0)
Supplement: Supplementary file 1 — Supplementary information [file 41413_2018_20_MOESM1_ESM.docx]

**Supplementary information**

**
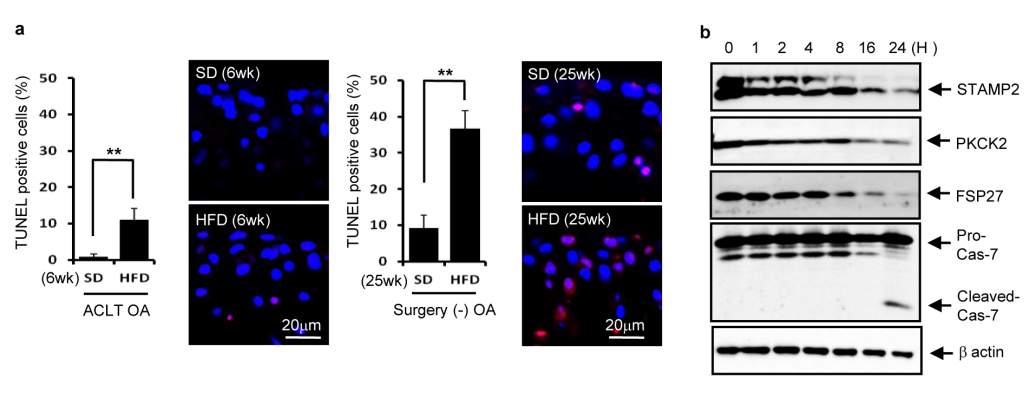
**

**Supplementary Figure 1.** (a) Quantification of TUNEL staining of the cartilage obtained from two OA models shows that the population of TUNEL positive articular chondrocytes was significantly higher in mice fed a HFD than in mice fed a SD. TUNEL-positive cells were quantified (*n* = 4). ** *P* < 0.01 versus mice fed a SD according to Scheffe’s test. The Pearson's correlation coefficient in ACLT model: 0.961 between PKCK2 and TUNEL. 0.878 between STAMP2 and TUNEL. 0.912 between FSP27 and TUNEL. The Pearson's correlation coefficient in surgery (-) OA (OA model without surgery) : 0.892 between PKCK2 and TUNEL. 0.795 between STAMP2 and TUNEL. 0.863 between FSP27 and TUNEL. (b) Time sequenced Western blots showing that PKCK2, STAMP2 and FSP27 proteins are downregulated at earlier time points than the time point of caspase activation (*n* = 4).
